# Cardiac-Specific Overexpression of ERRγ in Mice Induces Severe Heart Dysfunction and Early Lethality

**DOI:** 10.3390/ijms22158047

**Published:** 2021-07-28

**Authors:** Jaime Lasheras, Rosario Pardo, Marc Velilla, Marcos Poncelas, Núria Salvatella, Rafael Simó, Marisol Ruiz-Meana, Mònica Zamora, Josep A. Villena

**Affiliations:** 1 Laboratory of Metabolism and Obesity, Vall d’Hebron—Institut de Recerca, Universitat Autònoma de Barcelona, 08035 Barcelona, Spain; jaime.lasheras@vhir.org (J.L.); rosario.pardo@vhir.org (R.P.); marc.velilla@vhir.org (M.V.); salvatella.n@gmail.com (N.S.); 2 Laboratory of Experimental Cardiology, Vall d’Hebron—Institut de Recerca, Universitat Autònoma de Barcelona, 08035 Barcelona, Spain; poncelas@gmail.com (M.P.); marisol.ruizmeana@vhir.org (M.R.-M.); 3 Unit of Diabetes and Metabolism, Vall d’Hebron—Institut de Recerca, Universitat Autònoma de Barcelona, 08035 Barcelona, Spain; rafael.simo@vhir.org; 4 CIBER on Diabetes and Associated Metabolic Diseases (CIBERDEM), 28029 Madrid, Spain; 5 Institut d’Investigacions Biomediques August Pi i Sunyer, University of Barcelona, 08036 Barcelona, Spain

**Keywords:** cardiac dysfunction, estrogen-related receptors, transgenic mice

## Abstract

Proper cardiac function depends on the coordinated expression of multiple gene networks related to fuel utilization and mitochondrial ATP production, heart contraction, and ion transport. Key transcriptional regulators that regulate these gene networks have been identified. Among them, estrogen-related receptors (ERRs) have emerged as crucial modulators of cardiac function by regulating cellular metabolism and contraction machinery. Consistent with this role, lack of ERRα or ERRγ results in cardiac derangements that lead to functional maladaptation in response to increased workload. Interestingly, metabolic inflexibility associated with diabetic cardiomyopathy has been recently associated with increased mitochondrial fatty acid oxidation and expression of ERRγ, suggesting that sustained expression of this nuclear receptor could result in a cardiac pathogenic outcome. Here, we describe the generation of mice with cardiac-specific overexpression of ERRγ, which die at young ages due to heart failure. ERRγ transgenic mice show signs of dilated cardiomyopathy associated with cardiomyocyte hypertrophy, increased cell death, and fibrosis. Our results suggest that ERRγ could play a role in mediating cardiac pathogenic responses.

## 1. Introduction

Proper cardiac function depends on the coordinated expression of multiple gene networks that ensure functional adaption of the heart to changing demands on workload in response to physiological stimuli, such as exercise and pregnancy. The heart also adapts to the pathological stress imposed by increased hemodynamic loads, such as hypertension, via hypertrophic growth to maintain adequate contractility and blood supply to tissues. However, if such pathogenic stimuli persist, the heart may undergo a maladaptive remodeling that eventually leads to heart failure, a major cause of death and disability in modern society.

Programs related to fuel utilization and mitochondrial ATP production, as well as those associated with contraction, are critical for heart maturation and functional adaptation. During perinatal maturation of the heart, a metabolic shift from glucose utilization by means of glycolysis towards fatty acid oxidation (FAO) in mitochondria occurs to warrant the ATP production necessary to meet increased energy demands [1]. Such metabolic shift is accompanied by an intense mitochondriogenic process that endows cardiomyocytes with a remarkable number of mitochondria equipped with all the enzymatic machinery for efficient ATP production [2,3]. This metabolic reprograming during the perinatal period is paralleled by a complete remodeling of components of the contractile machinery characterized by a decrease in the fetal isoforms of sarcomeric proteins (i.e., β-MHC or TNNI1) and an increase of adult proteins isoforms (i.e., α-MHC, TNNI3, MYL2, etc.) [4]. In contrast, pathogenic cardiac remodeling in adult individuals is often associated with a reduction in mitochondrial FAO, increased glycolysis, and re-expression of fetal forms of sarcomeric proteins.

Numerous studies over the last two decades have contributed to uncover the network of key transcriptional regulators that tightly control metabolism and mitochondrial biogenesis during cardiac development, maturation, and physiological adaptation. Members of the peroxisome proliferator-activated receptor γ co-activator-1 (PGC-1) family of co-activators, PGC-1α and PGC-1β, have emerged as master regulators of cardiac energy metabolism. Although individual deletion of either *Ppargc1a* or *Ppargc1b* genes in rodent models does not lead to significant cardiac alterations due to the functional redundancy [5,6,7], their relevance in the control of cardiac energetics is demonstrated in mice simultaneously devoid of both PGC-1 co-activators specifically in cardiac cells, which die early after birth from heart failure [3]. Nonetheless, individual deletion of *Ppargc1a* or *Ppargc1b* accelerates heart failure in response to increased workload imposed by transverse aortic constriction [8,9].

Several hormone nuclear receptors have been identified as critical mediators of the action of PGC-1 co-activators on cardiac gene expression, including hormone thyroid receptor (TR) [10,11], peroxisome proliferator-activated receptor α (PPARα) [12,13], and estrogen-related receptors (ERRs). Interestingly, PPARα knockout mice show decreased FAO capacity and higher susceptibility to progressive deterioration of cardiac function during a work overload challenge [14]. A crucial role for ERRs in the adaptive changes of cardiac metabolism and function has also been demonstrated. Indeed, it has been shown that loss of ERRα leads to pathologic ventricular remodeling and dysfunction in response to chronic pressure overload. The development of this pathological condition in mice devoid of ERRα is associated with reduced expression of mitochondrial genes and impaired ATP production [15]. On the other hand, the role of ERRγ is especially relevant during cardiac development. Indeed, mice lacking ERRγ die early after birth from cardiac defects associated with severe alterations in mitochondrial oxidative metabolism [16]. Beyond bioenergetics, both ERRα and ERRγ have proven to be essential for cardiac contraction by regulating the expression of sarcomeric proteins during postnatal developmental maturation [17].

Whereas reduced expression of ERRs has been associated with impaired cardiac energetics and adaptability to imposed work overload, the potential protective role of increased ERRs activity has not been investigated. Here, we present a new mouse model of cardiac-specific overexpression of ERRγ. We show that sustained overexpression of ERRγ in cardiac tissue unexpectedly leads to premature death as a result of severe cardiac dysfunction.

## 2. Results

### 2.1. Transgenic Mice with Cardiac-Specific Overexpression of ERRγ Show Premature Lethality

**Figure 1:**
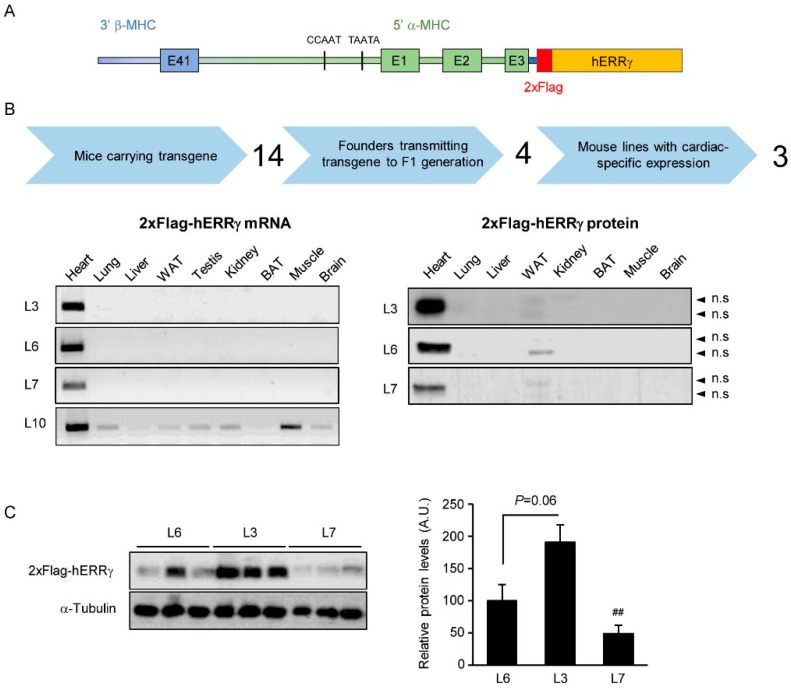
Generation of a mouse model of cardiac-specific overexpression of ERRγ (hERRγ-Tg). (**A**) A transgene that contains the cDNA encoding for a flag-tagged version of hERRγ under the control of the α-MHC promoter was used to generate mice that specifically overexpress ERRγ in the heart. (**B**) Three lines of hERRγ-Tg mice were obtained, first by selecting mice that specifically transmitted the transgene to their offspring, and then by ensuring that those mouse lines expressed the transgene specifically in the heart, both at the mRNA and protein level. (**C**) Western blot (left panel) comparing the expression levels of the Flag-hERRγ protein levels in hearts of the three transgenic lines obtained. Quantification of Flag-hERRγ protein expression in the heart of hERRγ-Tg mice (right panel). Data are mean ± SEM of three animals/line; ^##^ *p* = 0.01. # indicates the statistical significance of the comparison between L3 and L6; n.s. indicates nonspecific bands.

To investigate the metabolic and functional implications of sustained expression of high levels of ERRγ in heart, we generated transgenic mice that overexpress a flag-tagged version of human ERRγ under the heart-specific promoter of the α-MHC gene (Figure 1A), hereafter referred as hERRγ-Tg mice. Fourteen mice carrying the α-MHC-2xFlag-hERRγ transgene were obtained, but only four of these founders were able to transmit the transgene to the next generation. Analysis of the Flag-hERRγ mRNA expression by PCR revealed that only three of the transgenic mouse lines (named L3, L6, and L7) exhibited cardiac-specific expression of the transgene, whereas line L10 showed a preferential expression of the transgene in heart but detectable expression in almost all other tissues analyzed (Figure 1B, left panel). Cardiac-specific expression of the Flag-hERRγ protein was confirmed in lines L3, L6, and L7 by detecting the chimeric protein with a Flag antibody (Figure 1B, right panel). The three cardiac-specific transgenic lines exhibit different levels of Flag-hERRγ protein, L3 being the line with higher expression levels, L6 with intermediate expression levels, and L7 with lower expression levels of the protein (Figure 1C).

**Figure 2:**
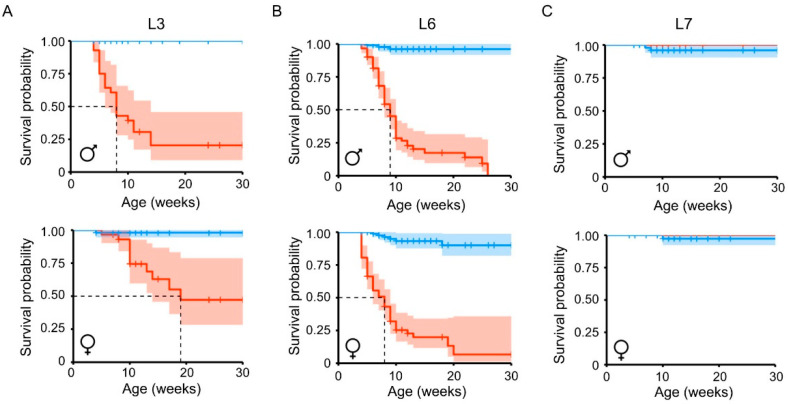
Survival estimates of Flag-hERRγ transgenic lines. Survival of both males and females from each of the three cardiac-specific hERRγ-Tg mouse transgenic lines was estimated by performing a Kaplan–Meier analysis for which the log rank test was used. L3 (**A**), L6 (**B**), and L7 (**C**); 80 to 202 animals per transgenic line and sex were used for the analysis.

Soon after establishing the three hERRγ-Tg lines, we observed an unusual lethality in young mice of lines L3 and L6, which expressed higher levels of Flag-hERRγ protein in the heart, but not in the low expresser line L7. Indeed, post-weaning analysis of mice survival over a period of 30 weeks showed that the median survival was severely decreased in L3 and L6 hERRγ-Tg mice, in both males and females (Figure 2). The estimated median survival was 8 weeks for males and 19 weeks for females of the L3 line (Figure 2A), and 9 weeks for both males and females of the L6 line (Figure 2B). No differences in the survival rate between Wt and hERRγ-Tg mice of the low expresser L7 line were observed (Figure 2C).

### 2.2. Mice Overexpressing High Levels of ERRγ Exhibit Cardiac Functional Defects

**Figure 3:**
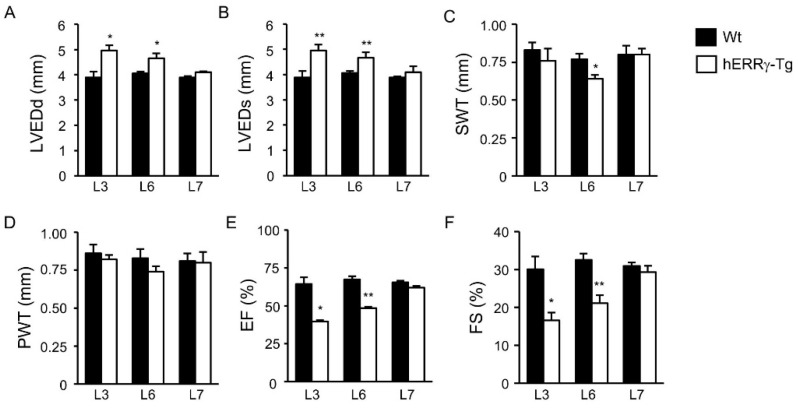
Structural and functional alterations in hearts of hERRγ-Tg mice. Cardiac dimensions and function in 8–10-week-old hERRγ-Tg mice were assessed by transthoracic M-mode and two-dimensional echocardiography. (**A**) LVEDd: left ventricular end-diastolic internal diameter; (**B**) LVEDs: left ventricular end-systolic internal diameter; (**C**) SWT: interventricular septum wall thickness; (**D**) PWT: posterior wall thickness; (**E**), EF: ejection fraction; (**F**) FS: fractional shortening). Data are mean ± SEM of 3–4 animals/group; * *p* = 0.05; ** *p* = 0.01.

Because the overexpression of hERRγ in Tg mice was restricted to the heart, we investigated if the premature lethality observed was due to a cardiac defect. Echocardiographic analysis of male mice revealed significant morphological alterations in hERRγ-Tg mice of the L3 and L6 lines (Figure 3). Indeed, a substantial increase in the end-diastolic left ventricular internal diameter (LVEDd) and end-systolic left ventricular internal diameter (LVEDs) was observed in mice of lines L3 and L6 (Figure 3A,B), which show the highest levels of Flag-hERRγ in the heart. Moreover, a significant decrease in the interventricular septum wall thickness (SWT) was only observed in mice of the L6 line (Figure 3C), whereas no changes were observed in the thickness of the heart posterior wall (PWT) in any of the transgenic lines (Figure 3D). The structural alterations observed in lines L3 and L6 resulted in an overt decline of cardiac function, as evidenced by reduced left ventricular ejection fraction (EF) and fractional shortening (FS) (Figure 3E,F), tightly correlating with the expression levels of the Flag-hERRγ protein. No evidence of structural or functional alterations was found in the L7 line, which show the lowest expression levels of the transgene among all the transgenic lines generated.

**Figure 4:**
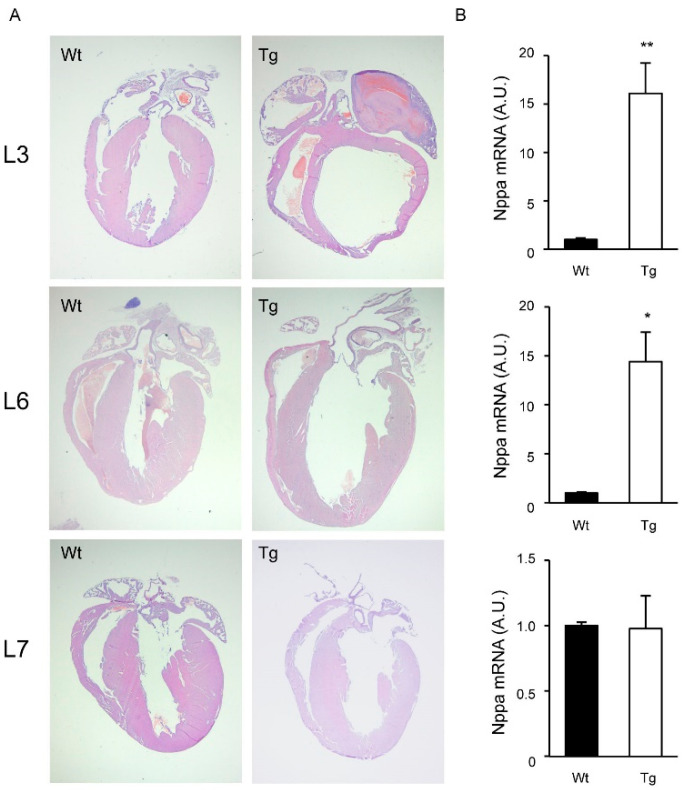
Morphological abnormalities in hearts of hERRγ-Tg mice. (**A**) Histological sections of hERRγ-Tg mouse lines L3, L6, and L7 stained with hematoxilin/eosin. (**B**) mRNA expression of *Nppa* was determined by real-time quantitative PCR in cardiac tissue from hERRγ-Tg mice. Data are mean ± SEM of 3–4 animals/group; * *p* = 0.05; ** *p* = 0.01.

Morphological inspection of hERRγ-Tg mice revealed enlarged hearts in those animals from lines L3 and L6, but not in those of line L7. Histological analysis of hearts confirmed the echocardiographic results. Indeed, compared to their Wt littermates, hERRγ-Tg mice from line L3 exhibited enlarged ventricular cavities along with marked thinning of the ventricular walls and interventricular septum, indicative of pathological cardiac dilation (Figure 4A). Although less dramatic, a similar phenotype was observed in histological preparations of hERRγ-Tg mice from line L6. In contrast, in agreement with the low expression levels of Flag-hERRγ and the echocardiographic results, no structural differences were observed between hERRγ-Tg mice and Wt littermates of the line L7. Consistent with the functional decline observed, a dramatic increase in the expression of atrial natriuretic peptide (*Nppa*) was detected in hearts of transgenic mice from the L3 and L6 lines, but not the L7 line (Figure 4B).

### 2.3. ERRγ Cardiac Overexpression Is Associated with Cardiomyocyte Hypertrophy, Apoptosis, Fibrosis, and Altered Gene Expression

**Figure 5:**
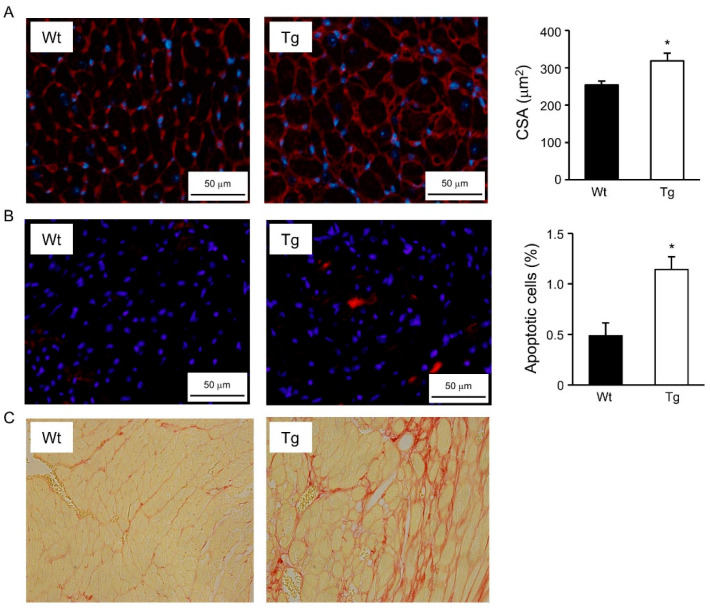
Cellular alterations in hearts of L6 hERRγ-Tg mice. (**A**) Cardiomyocyte cross-sectional area (CSA) was determined using the ImageJ software after staining heart sections with wheat germ agglutinin (WGA) conjugated with FITC. (**B**) Apoptosis was estimated by immunodetection with active caspase-3 (red). (**C**) Cardiac fibrosis was detected with the sirius red staining. Data are mean ± SEM of 3–4 animals/group; * *p* = 0.05.

To investigate the cellular effects of ERRγ overexpression potentially involved in cardiac dysfunction, we focused on line L6, which shows intermediate levels of the Flag-hERRγ protein but still develops an overt cardiac phenotype. Morphometric analysis revealed an increase in the cross-sectional area of ventricular cardiomyocytes from 7 to 10-week-old hERRγ-Tg mice, compared to their Wt littermates (Figure 5A). In addition, apoptosis was increased in hearts of hERRγ-Tg mice, as revealed by the abundance of active caspase-3 positive cells (Figure 5B). Moreover, cardiac dysfunction in hERRγ-Tg mice was also associated with increased fibrosis, as demonstrated by sirius red staining of histological sections of hearts (Figure 5C).

**Figure 6:**
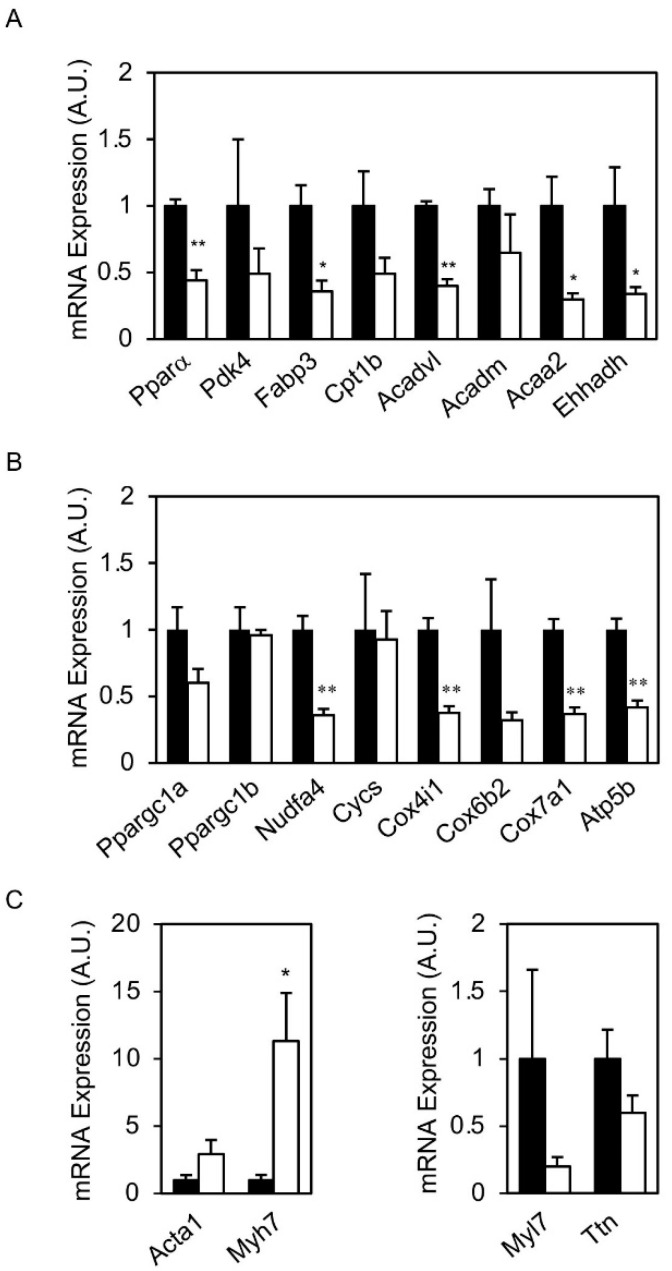
Expression of ERRγ target genes in adult L6 hERRγ-Tg mice. mRNA expression of genes related to lipid metabolism (**A**), OxPhos system (**B**), and sarcomeric proteins (**C**) was determined by real-time quantitative PCR. Data are mean ± SEM of 3–4 animals/group; * *p* = 0.05; ** *p* = 0.01.

ERRγ has been shown to regulate the expression of genes related to oxidative metabolism, particularly those related to mitochondrial biogenesis and function, as well as genes related to the contractile function of cardiac cells. Therefore, expression of ERRγ target genes was assessed in hearts of young hERRγ-Tg and Wt mice from line L6. Surprisingly, expression of mitochondrial genes related to FAO (i.e., *Acadvl, Acaa2, ehhadh* and *Cpt1b*) (Figure 6A) and the OxPhos system (i.e., *Atp5b*, *Cox4i1, Cox7a1* and *Ndufa4*) (Figure 6B) were severely downregulated in hearts of hERRγ-Tg mice. Similarly, genes related to other aspects of lipid metabolism (i.e., *Ppara* and *Fabp3*) were also decreased (Figure 6A). Furthermore, we observed an exacerbated increase in the expression of genes encoding for the fetal isoforms of myosin heavy chain (*Myh7*) and actin gene (*Acta1*), whereas adult isoforms of contractile proteins, such as titin (*Ttn*), were downregulated (Figure 6C).

**Figure 7:**
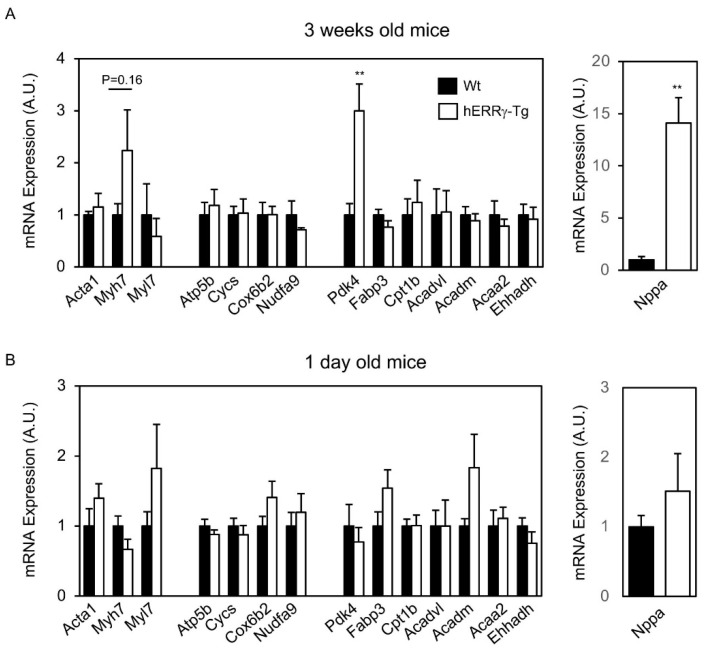
Expression of ERRγ target genes in young L6 hERRγ-Tg mice. mRNA expression of genes related to sarcomeric proteins, OxPhos system, and lipid metabolism, as well as *Nppa,* was determined by real-time quantitative PCR in 3-week-old (**A**) and 1-day-old (**B**) hERRγ-Tg mice and Wt littermates. Data are mean ± SEM of 3–5 animals/group; ** *p* = 0.01.

In order to shed some light on whether the gene expression profile observed in adult L6 hERRγ-Tg mice is the direct result of ERRγ overexpression or simply secondary to heart failure, we analyzed expression of ERRγ-target genes in younger animals. Interestingly, at 3 weeks of age, hERRγ-Tg mice show expression of OxPhos and FAO genes similar to that of Wt mice (Figure 7A). Still, at this age, hERRγ-Tg mice exhibit very high levels of *Nppa* and *Myh7*, indicating some degree of cardiac functional alteration. On the other hand, hearts of 1-day-old mice show a gene expression profile that is, at least in part, compatible with increased ERRγ transcriptional activity. Indeed, expression of some *bona fide* ERRγ target genes, such as those involved in lipid metabolism (i.e., *Fabp3*, *Acadm*) and OxPhos (i.e., *Cox6b2*), appears mildly induced in hearts of hERRγ-Tγ mice, although differences do not reach statistical significance (Figure 7B). Moreover, hearts from 1-day-old mice show higher levels of *Myl7* (myosin light chain 7) mRNA, which is expressed in hearts of healthy individuals, whereas the expression of *Nppa* is almost the same as in Wt mice. Altogether, these results suggest that at early developmental stages, ERRγ overexpression induces a gene expression profile characterized by the induction of genes involved in oxidative metabolism. However, as hERRγ-Tg mice get older, heart failure develops and leads to a reduction in the expression of mitochondrial OxPhos and FAO genes, at the same time as it alters the proper expression of genes encoding for contractile proteins.

## 3. Discussion

The results obtained in our study indicate that sustained cardiac overexpression of ERRγ is sufficient to induce a severe cardiomyopathic phenotype that leads to lethality in young transgenic mice. Several studies have demonstrated that members of the ERR family of orphan nuclear receptors play an important role in the heart, being necessary for proper cardiac maturation and functional adaptation to work overload by regulating the expression of genetic programs related to energy metabolism and contraction [15,16,17]. In this sense, whole-body deletion of ERRγ results in premature death within 48 h after birth [16]. These mice exhibit an abnormal ventricular depolarization and delayed cardiomyocyte repolarization, in part due to reduced expression of the sodium channel SCN5A. In addition to altered current conduction, ERRγ null mice also show a decrease in the expression of mitochondrial genes, particularly of genes related to the OxPhos system. Therefore, the limited ATP production resulting from impaired mitochondrial function may have also contributed to the cardiac dysfunction observed in null neonates [16]. However, whether the neonatal lethality of whole-body ERRγ knockout mice is the direct result of the severe cardiac alterations observed in these mice has been questioned by studies using rodent models with specific deletion of the *Esrrg* gene in the heart. Indeed, cardiac-specific ERRγ knockout mice, despite showing a decrease in the expression of mitochondrial genes, exhibit normal survival rates and no gross cardiac alterations, indicating that postnatal cardiac loss of ERRγ does not bring about a lethal phenotype [18]. In contrast to ERRγ, ERRα seems to be dispensable for proper cardiac maturation, and ERRα null mice develop normally, without any apparent cardiac defect [19]. Nevertheless, loss of ERRα accelerates the functional and energetic defects associated with increased pressure overload of adult mice [15], pointing towards a relevant role of ERRα in cardiac adaptation to increased workload. Altogether, these findings rather suggest a functional redundancy between ERRα and ERRγ. Such functional compensation is in agreement with the high degree of overlap existing between the target genes of both ERRs [20] and recent studies showing that simultaneous postnatal loss of ERRα and ERRγ in the heart induce severe alterations in cardiac function, tightly associated with altered expression of mitochondrial genes, reduced mitochondrial biogenesis, and altered expression of contractile proteins [17,18].

Considering the deleterious effects described as a result of cardiac loss of ERRγ, one would expect that overexpression of ERRγ in the heart should either cause no harm to mice, or even improve cardiac metabolism by increasing the expression of genes encoding for mitochondrial or adult contractile proteins. To our surprise, sustained cardiac overexpression of ERRγ led to a severe cardiomyopathic phenotype characterized by severe structural and metabolic derangements, including reduced expression of mitochondrial genes and increased expression of the fetal forms of contractile proteins. Considering that genes encoding for mitochondrial and adult sarcomeric proteins are well-known targets of ERRγ, the suppressed expression of these genes seems to be secondary to the development of cardiomyopathy, rather than the direct regulatory effect of ERRγ on gene expression. In this regard, it is noteworthy to remark that the pathophysiology of heart failure is characterized by a metabolic remodeling distinguished by a depression of energy metabolism, in particular, reduced FAO and OxPhos gene expression and impaired mitochondrial biogenesis [1], similar to what occurs in our hERRγ-Tg model. Also supporting the notion that reduced expression of genes related to oxidative metabolism in adult hERRγ-Tg mice is secondary to the progression of heart failure, the gene expression profile in younger transgenic mice is compatible with an expression profile resulting from increased ERRγ transcriptional activity, which includes increased mRNA levels of genes involved in mitochondrial oxidative phosphorylation, lipid metabolism, and postnatal contractile machinery. Still, we cannot absolutely rule out the possibility that the decrease in the expression of ERRγ *bona fide* targets observed in adult hERRγ-Tg mice was the result of co-activator squelching due to the very high levels of Flag-hERRγ protein.

The hERRγ-Tg mice displayed ventricular dilation and heart failure, demonstrating that sustained increase in the levels of ERRγ protein is sufficient to induce cardiac dysfunction. In support of this hypothesis, we have recently reported that heart dysfunction in obese diabetic *db/db* mice is characterized by a gene expression profile featuring increased expression of OxPhos, FAO, and lipid metabolism genes, as well as alterations in the expression of contractile proteins, including an increase in the expression of fetal isoforms of sarcomeric proteins [21]. Interestingly, a computational analysis conducted in order to find potential transcriptional regulators responsible for this expression profile found ERRγ as a major regulatory node [21]. The capacity of ERRγ to drive the expression profile and metabolic derangements found in hearts of *db/db* mice was demonstrated in cultured neonatal cardiomyocytes, in which adenoviral-mediated overexpression of a Flag-hERRγ chimeric protein was sufficient to induce cardiomyocyte hypertrophy, increase mitochondrial gene expression, and enhance FAO [21]. Similar to our studies, Kwon and collaborators reported that ERRγ overexpression in cultured cardiomyocytes leads to cellular hypertrophy in a Gata-4-dependent manner [22]. Importantly, this study also reported that patients with hypertrophic cardiomyopathy exhibit high content of ERRγ protein in the heart [22], supporting the notion that elevated levels of ERRγ could be associated with cardiac disease also in humans. 

ERRγ is not the only transcriptional regulator that plays a dual role in cardiac pathophysiology. Indeed, PPARα has been shown to have a protective role by maintaining normal cardiac function under basal conditions and preventing hypertrophic growth in response to pressure overload [12,13]. However, elevated cardiac levels of PPARα have been found in a pathological context, such as in diabetic individuals [23,24,25]. It has been suggested that elevated levels of PPARα in diabetic hearts contribute to the development of metabolic and functional alterations that characterize diabetic cardiomyopathy by promoting cardiac steatosis and lipotoxicity through the activation of the expression of genes related to fatty acid uptake and oxidation. The pathogenic activity of PPARα has been unequivocally demonstrated in transgenic mice with cardiac-specific overexpression of PPARα, which, similar to what we have described for ERRγ, develop ventricular hypertrophy and systolic dysfunction [24,25] and exhibit delayed postischemic recovery of cardiac function [26]. Analogously, chronic treatment with PPARα agonists impairs heart function, at least in part, by attenuating cardiomyocyte respiratory capacity [27]. A dual, either protective or pathogenic, role has also been described for PGC-1α, a transcriptional co-activator of many nuclear receptors, including PPARα and ERRγ [28]. Although mice lacking PGC-1α do not exhibit any gross cardiac morphological or functional alteration under basal non-stimulated conditions [5], when subjected to increase workload by transverse aortic constriction, these mice develop significant heart hypertrophy and accelerated heart failure [8]. However, cardiac overexpression of PGC-1α, far from improving cardiac function, has been shown to lead to severe myocardiopathy and early lethality [29,30,31]. Similar to PGC-1α, cardiac deficiency of PGC-1β leads to heart hypertrophy and functional failure only when mice are subjected to pressure overload [9]. As for ERRs, the redundant role of PGC-1α and PGC-1β in controlling mitochondria biogenesis in cardiomyocytes could have contributed to the absence of a cardiac phenotype under basal conditions. This notion is supported by the early lethality resulting from the severely impaired contractile function exhibited by mice with simultaneous deficiency of both PGC-1α and PGC-1β in the heart [32]. The effects of sustained overexpression of PGC-1β in the heart have not been yet elucidated.

The contribution of ERRγ to human cardiac development, function, or pathogenesis remains to be precisely defined. Whereas a study reported that the expression of ERRγ, as well as that of ERRα and PGC-1α, is decreased in cardiac tissue of patients with heart failure [33], another described an increase in ERRγ protein in patients with cardiac hypertrophy [22]. These apparently contradictory data are somehow in line with the data obtained from genetically engineered mouse models, in which reduced expression of ERRγ accelerated the progression of cardiac dysfunction during embryonic development or in response to increased cardiac overload [16,17,18], whereas, as demonstrated in the present study, sustained overexpression induces severe heart failure. In view of these findings, the use of drugs that modulate ERRγ activity for the treatment of cardiac diseases in humans seems plausible. In support of this notion, it has been shown that treatment of cultured cardiomyocytes with the ERRγ inverse agonist GSK-5182 prevents hypertrophy induced by phenylephrine [22]. Still, the therapeutic use of ERRγ modulators in humans—and animal models—is hampered by their limited bioavailability and specificity. Additionally, an important aspect that needs to be taken into account when considering the use of ERRγ synthetic ligands as therapeutic drugs is to limit their use within a defined therapeutic window, since both sustained activation or inhibition of ERRγ could lead to cardiac dysfunction.

In summary, despite the crucial role of ERRγ in cardiac development and functional adaption to increased workload, our study shows that sustained elevated levels of ERRγ, far from improving cardiac function, have a detrimental effect on the heart, promoting the development of cardiomyopathy and heart failure. Our results, together with evidence provided by other studies, point towards the axis PGC-1/ERR/PPARα as a crucial regulatory circuit whose fine-tuning is essential for proper cardiac function. Alterations of this regulatory axis that result in either a sustained reduction or increase in the transcriptional activity of any of its components can lead to cardiac dysfunction. Taken together, these studies indicate that pharmacological modulation of the PGC-1/ERR/PPARα should be carefully considered, since either sustained activation or inhibition may lead to undesirable harmful cardiac effects.

## 4. Materials and Methods

### 4.1. Generation of Cardiac-Specific ERRγ Transgenic Mice

To generate transgenic mice with cardiac overexpression of ERRγ, the coding sequence of human ERRγ (hERRγ) containing two Flag epitopes in the 5′ region were amplified by PCR using a pcDNA3.1-2xFlag-hERRγ vector as a template (described in [21]) and primers 5′-ACTGTCGACGCCACCATGGATTACAAGGATGACGACGAT-3′ (forward) and 5′-ACTGTCGACTTAGCAGACCTTGGCCTCAAACATTTC-3′ (reverse). The resulting fragment was digested with SalI restriction enzyme and cloned into the SalI site of the pBSII-SK-αMHC that contains a 5.7 kb of mouse genomic DNA comprising the 3′ end of the β-cardiac MHC gene, the intergenic region, and the 5′ end of the α-cardiac MHC gene, ensuring cardiac-specific expression of the 2xFlag-hERRγ cDNA [34]. A ≈7.4 kb BamHI digestion fragment was obtained, purified, and microinjected into the pronuclei of fertilized mouse eggs (C57BL/6J mice). Embryos were implanted into pseudo-pregnant foster females at the Mouse Mutant Core Facility of the Institute for Research in Biomedicine (IRB, Barcelona, Spain).

Transgenic mice were identified by PCR. For this, DNA was purified from tail clips and used to amplify a 537 bp fragment of the transgene using the following primers: 5′-GCAGGGAAGTGGTGGTGTAGG-3′ (forward) and 5′-ACCCAGAAGCGATGTCACCAC-3′ (reverse). Mice were housed in a temperature-controlled environment at 21 °C, subjected to a 12 h/12 h light/dark cycle and fed a standard diet (2018 Tecklad Global 18% Protein Rodent Diet, 18 Kcal from fat, Harlan Laboratories).

### 4.2. Gene Expression Analysis

For gene expression analysis, RNA was isolated from heart or other tissues with Trizol (Thermo Fisher Scientific, Waltham, MA, USA) according to the manufacturer’s instructions. cDNA was synthesized from 400 ng of RNA with SuperScript II reverse transcriptase (Thermo Fisher Scientific, Waltham, MA, USA) and oligo dT primers. Gene expression was assessed by real-time quantitative PCR (qPCR) using gene-specific primers and SYBR green (Thermo Fisher Scientific, Waltham, MA, USA) in an ABI PRISM 7500 Sequence Detection System (Thermo Fisher Scientific, Waltham, MA, USA). Relative mRNA expression was calculated according to the 2^−∆∆CT^ threshold cycle method, using cyclophilin A as a reference gene, as previously described [35].

The tissue-specific expression of the 2xFlag-hERRγ transgene was achieved by conventional PCR using cDNA obtained from total RNA by reverse transcription as a template and specific primers 5′-GGTTCAGCCAGCCAAAAAGCC-3′ (forward) and 5′-CAGGGACAGCGTGGAGAAGCC-3′ (reverse) that allow the detection of hERRγ. The resulting 204 bp amplicon was resolved in a 2% agarose gel.

### 4.3. Western Blot

Protein extracts from hearts and other tissues were prepared in homogenization buffer (25 mM Tris-HCl pH = 7.5, 150 mM NaCl, 1% sodium deoxycholate, 1% Nonidet P-40, 0.1% Triton X-100, 1% SDS, 10 mM NaF, 1 mM Na_3_VO_4_) supplemented with protease inhibitors. Forty μg of proteins were resolved by 12% SDS-PAGE, transferred to a PVDF membrane, and probed with specific antibodies against Flag epitope (Merck, Darmstat, Germany) or α-tubulin (Cell Signaling Technology, Danvers, MA, USA).

### 4.4. Histological Analysis

For histological analysis, the hearts were rapidly removed, washed in saline, and fixed in formalin overnight. After sequential dehydration in ethanol solutions of increasing concentrations, whole hearts were embedded in paraffin. Then, 5–8 μm heart sections were obtained and stained with hematoxylin/eosin for gross morphological examination. To detect cardiac fibrosis, heart sections were stained with sirius red, which specifically stains collagen fibers.

To measure the cardiomyocyte cross-sectional area, paraffin sections of hearts were stained with wheat germ agglutinin conjugated with TRITC and DAPI. Pictures of three different fields of each heart were randomly taken (*n* = 3 mice/group), and the area of at least 900 cells/heart was measured with the ImageJ Software.

### 4.5. Apoptosis

Cell apoptosis was determined in frozen sections of mouse hearts by immunohistochemistry, as previously described [21]. Briefly, after dissection, hearts were rapidly frozen in OCT compound, and 8–10 μm sections were obtained in a Leica CM-3050-S Cryostat (Leica Microsystems, Wetzlar, Germany). To detect apoptotic cells, sections were first incubated with a rabbit polyclonal antibody against active caspase-3 (Abcam, Cambridge, UK), and subsequently incubated with an anti-rabbit goat secondary antibody conjugated with the Alexa Fluor 594 dye (Abcam, Cambridge, UK) and DAPI. To quantify the number of apoptotic cells, 8–10 fields from each heart were randomly taken (at least 800 cells/heart, *n* = 3 mice/group), and total and apoptotic cells counted.

### 4.6. Echocardiography

Cardiac function and dimensions were assessed by transthoracic M-mode and two-dimensional echocardiography in mice under light anesthesia (0.5–1% isofluorane) using a Vivid Q portable ultrasound system with an ILS 12 MHz transducer (GE Healthcare, Bosston, MA, USA). M-mode images of four different cardiac cycles from each animal were analyzed to assess cardiac function. The ejection fraction (EF), left ventricular end-diastolic internal diameter (LVEDd), left ventricular end-systolic internal diameter (LVEDs), interventricular septum wall thickness (SWT), posterior wall thickness (PWT), and fractional shortening (FS) were blindly measured.

### 4.7. Statistical Analysis

Results are expressed as mean ± SEM. The statistical significance of differences between experimental groups was assessed using an unpaired *Student’s t-test* or a one-way analysis of the variance (ANOVA) followed by *Tukey post hoc test*. For the Kaplan–Meier survival analysis, the *log rank test* was used. Differences were considered significant when *p* < 0.05.

## Data Availability

The data presented in this study are available on request from the corresponding author.
